# The Organization and Control of Intra-Limb Anticipatory Postural Adjustments and Their Role in Movement Performance

**DOI:** 10.3389/fnhum.2016.00525

**Published:** 2016-10-19

**Authors:** Paolo Cavallari, Francesco Bolzoni, Carlo Bruttini, Roberto Esposti

**Affiliations:** Human Motor Control and Posture Lab, Section Human Physiology of the Department of Pathophysiology and Transplantation, Università degli Studi di MilanoMilan, Italy

**Keywords:** intra-limb APAs, precision, motor control, postural control, posturo-focal integration, human

## Abstract

Anticipatory Postural Adjustments (APAs) are commonly described as unconscious muscular activities aimed to counterbalance the perturbation caused by the primary movement, so as to ensure the whole-body balance, as well as contributing to initiate the displacement of the body center of mass when starting gait or whole-body reaching movements. These activities usually create one or more fixation chains which spread over several muscles of different limbs, and may be thus called *inter-limb APAs*. However, it has been reported that APAs also precede voluntary movements involving tiny masses, like a flexion/extension of the wrist or even a brisk flexion of the index-finger. In particular, such movements are preceded by an *intra-limb* APA chain, that involves muscles acting on the proximal joints. Considering the small mass of the moving segments, it is unlikely that the ensuing perturbation could threaten the whole-body balance, so that it is interesting to enquire the physiological role of *intra-limb* APAs and their organization and control compared to *inter-limb* APAs. This review is focused on *intra-limb* APAs and highlights a strict correspondence in their behavior and temporal/spatial organization with respect to *inter-limb* APAs. Hence it is suggested that both are manifestations of the same phenomenon. Particular emphasis is given to *intra-limb* APAs preceding index-finger flexion, because their relatively simple biomechanics and the fact that muscular actions were limited to a single arm allowed peculiar investigations, leading to important conclusions. Indeed, such paradigm provided evidence that by granting a proper fixation of those body segments proximal to the moving one APAs are involved in refining movement precision, and also that APAs and prime mover activation are driven by a shared motor command.

## Introduction

Anticipatory Postural Adjustments (APAs) are commonly described as unconscious muscular activities aiming to maintain the equilibrium of the whole body during various voluntary motor performances (for a review see Massion, 1992; Bouisset and Do, 2008), but they are also known to contribute to initiate the displacement of the body center of mass when starting gait (Brenière et al., 1987) or whole body reaching movements (Stapley et al., 1998, 1999). However, the importance of APAs is also apparent when considering motion of one single limb (e.g., Belen’kii et al., 1967 for the upper limb; Alexeief and Naidel, 1972 for the lower limb). In the latter context, the main goal of APAs is to minimize the changes in the body center of mass, in order to keep its projection within the support area, and to counteract the self-initiated postural perturbation. These APAs usually spread over several muscles of different limbs, creating one or more fixation chains. For this reason they may be called *inter-limb APA*.

The role of APAs may look less obvious when considering a movement involving little masses, which are not supposed to threaten the whole body postural stability. In this regard, Aoki (1991) reported a pattern of muscular activity in various arm muscles about 50–60 ms before a rapid wrist movement, and showed that this activity is distributed according to the direction of the movement in space. Moreover, Caronni and Cavallari (2009a) found that during index-finger tapping, in which the moving mass is even smaller, an anticipatory postural chain develops in several upper-limb and trunk muscles. These activities, named *intra-limb APAs* since they are distributed to muscles of the same limb in which the movement occurs, precede the onset of the voluntary movement, are polarized according to the task direction in space and adapt to changes in the postural requirement of the task. In other words, they share the same behavioral properties of *inter-limb APA*, as it will be shown as the *first topic* of the present review.

Nevertheless, it is difficult to envisage a postural role for *intra-limb APAs* in the common sense of its acceptation, i.e., preserving the whole body equilibrium or counteracting a self-initiated perturbation that could threaten the body posture. This *intra-limb* APA pattern seems in fact organized so as to preserve the *local equilibrium* of the limb, which, in a behavioral perspective represents a basic requirement for performing precise movements. The involvement of *intra-limb* APAs in setting movement precision will be treated as the *second topic*.

Moreover, the *finger tap* experimental model allowed us to disclose some key aspects of the central organization of voluntary movement and postural actions, aspects that would hardly be revealed by using the classical *whole body* experimental paradigm. Thus, the *last topic* of this review will be about the organization of voluntary and postural actions, either as two different central commands (a focal and a postural component), as classically proposed (Babinski, 1899; Thomas, 1940; Hess, 1943; Gelfand et al., 1966; Cordo and Nashner, 1982; Brown and Frank, 1987) or as a unique motor command as suggested by more recent evidences (Aruin and Latash, 1995; Caronni and Cavallari, 2009b; Petersen et al., 2009; Leonard et al., 2011).

### Brief Historical Background on Posture and Voluntary Movement

To the best of our knowledge, the first to describe the rules governing postural and voluntary movement control was Leonardo da Vinci (1452–1519), in his *Libro A, Trattato della Pittura*, which is now conserved within the *Codex Urbinas Latinus*. Leonardo wrote: “*I say that if a motionless figure is poised on his feet, and his arm is extended in front of the chest, he will throw backward as much natural weight as the natural and accidental weight which he thrusts forward. And I say the same of each part that projects more than usual beyond the whole*”. Moreover, Leonardo stated: “*Never will a weight be lifted and carried by a man, without his extending outside himself more than as much weight as that which he wishes to lift, and he thrust it on the side opposite the one where he lifts the weight*”. A couple of centuries later, Giovanni Alfonso Borrelli (1608–1679) followed the pathway of Leonardo in his *De Motu Animalium*, where he described some principles that govern the voluntary movement. In particular, Borelli considered the skeleton as multi-linked system of levers, which can maintain the body balance as long as the center of mass falls within the support base.

After the observation of Leonardo da Vinci and Giovanni Borelli, we had to wait until the end of the 19th century to first clinically observe the importance of correct postural tailoring during voluntary movements in order to execute a successful motor act. Joseph Babinski (1857–1932), in his *De l’asynergie cérebelleuse*, first described the lack of harmonious synergies in cerebellar patients; in particular, he observed the forward displacement of the knees and hips to compensate for the backward displacement of the trunk, neck and head when asking a subject to look upward. Babinski also observed that cerebellar patients, who were asked to perform the same *looking upward* task, were unable to coordinate the upper part of their body to their hip and lower limb, so that they usually fell. However, Babinski did not analyze the temporal relationship between the postural adjustments and the voluntary movement, and therefore he lacked to observe that also an anticipatory postural control, adjusted in a feed-forward manner, is needed when performing a voluntary movement.

In the 20th century, studies about the relationship between posture and voluntary movement stood on the shoulders of three giants of movement physiology: Charles Sherrington (1857–1952, Nobel Prize 1932), Walter Hess (1881–1973, Nobel Prize 1949) and Nikolai Bernstein (1896–1966, Stalin Prize for Science 1948). Sherrington sustained the idea of a dual coordinated control system: one for movement and one for posture. This idea was in agreement with the proposals of Hess, who held the view that without anticipation of postural adaptations (a component of his ereismatic-supporting function system), goal-directed movements were doomed to failure. On the other hand, Bernstein was the first to propose a unique motor command for both posture and voluntary movement: “*movements are not chains of details but structures which are differentiated into details; they are structurally whole*” (Bernstein, 1967). However his ideas did not reach western scientists until several years after his death since his articles and books were only recently translated into English.

## Shared Behavioral Properties of Inter and Intra-Limb APAs

This review deals with *intra-limb* APA, since many other reviews (see “Introduction” Section) have been already devoted to all the features characterizing *inter-limb* APAs. However, it is important to briefly recall (see “Inter-Limb APAs” Section) those specific aspects of *inter-limb* APAs which can be directly compared with *intra-limb* APAs. All other features, like the effects of development, aging and fatigue, extensively studied in *inter-* but not in *intra-limb* APAs, have been thus omitted.

With regard to literature selection, this is a narrative more than a systematic meta-analysis review because in the last decade the *intra-limb* APAs has been mainly treated by our research group (for older studies see “Intra-Limb APAs” Section). However, to avoid missing important literature, *Pubmed*® and *Web of Science*® databases were inquired, by searching for *APAs* or *Anticipatory Postural Action(s)* and verifying item by item whether it was dealing with *inter-* and/or *intra-limb* APAs.

### Inter-Limb APAs

These are the most frequently studied APAs. For example, when we intend to move a segment of the upper or the lower limb, a chain of muscular actions develops from that moving segment to the nearest fixation point. These actions precede the primary movement (in the range of tenths ms) and in many cases also the prime mover recruitment, the larger advance being observed in the muscles acting near the fixation point.

These *inter-limb* APAs have been documented for various movements such as: shoulder flexion and extension (Belen’kii et al., 1967; Lee, 1980; Clément et al., 1984; Horak et al., 1984; Bouisset and Zattara, 1987; Maki, 1993 in adults; Riach and Hayes, 1990 in children), shoulder lateral abduction (Aruin and Latash, 1995; Vernazza et al., 1996), elbow flexion (Friedli et al., 1984) and similar movements of the lower limb (Rogers and Pai, 1990; Do et al., 1991; Mouchnino et al., 1991; Nouillot et al., 1992). APAs also accompany movement involving the trunk, when bending it (Oddsson and Thorstensson, 1986; Crenna et al., 1987; Pedotti et al., 1989), during whole body reaching (Stapley et al., 1998), rising up on tiptoe (Houtz and Fischer, 1961; Lipshits et al., 1981; Nardone and Schieppati, 1988) and rocking on the heels (Nardone and Schieppati, 1988). In some experimental situations, it was asked to push or pull a handle with the upper limb, both in normal subjects (Cordo and Nashner, 1982; Lee et al., 1987) and in patients with Parkinson disease (Dick et al., 1986). *Inter-limb APAs* are also associated with movements involving a bimanual load-lifting task, when participants hold an object in one hand and the object is voluntarily lifted by the same person (active lifting) or by an experimenter (passive lifting). In the first case, the voluntary activation of the lifting hand is synchronous with an inhibitory APA in the contralateral biceps brachii (BB), thus avoiding an uncontrolled flexion of the elbow (Hugon et al., 1982) and preserving the upper limb posture. On the contrary, when the object is unexpectedly lifted by the experimenter, the BB inhibition starts about 50 ms after the unloading, showing that is it should have a reflex origin (postural reflex). The described experiment, known as “*the barman task*” allowed to make two conclusions: first, even a movement that does not lead to a whole body imbalance is preceded by APAs, aimed at preserving the single segment’s balance. Second, APAs may develop also in muscles that are not usually considered as *postural muscles*, such as muscles in the upper-limb. Moreover, since the degree of elbow rotation was shown to be lower when the subject unloaded the forearm by himself, the APA mechanism showed a greater efficacy in limb stabilization vs. the reflex mechanisms. The involvement of APAs in controlling the segmental stability was also confirmed for movements affecting the whole body balance (Patla et al., 2002).

Classically, APAs are known to be tuned depending on several kinematic aspects of the primary movement; specifically, the amplitude (Aruin and Shiratori, 2004), the speed (Horak et al., 1984; Shiratori and Aruin, 2007), the movement direction (Aruin and Latash, 1995; Pozzo et al., 2002) and also the mass of the moving segment (Friedli et al., 1984). Moreover, an increase of APAs was shown when the voluntary movement was performed against resistance (Baldissera et al., 2008).

In addition, the CNS is able to adapt *inter-limb APAs* to changes in the postural demand of the motor task (Belen’kii et al., 1967; Cordo and Nashner, 1982; Aruin and Shiratori, 2004; Shiratori and Aruin, 2007). Clear signs of this adaptative process have been observed even after the very first movement trial (Hall et al., 2010), although they usually develop within few movement repetitions.

Another feature of the APAs is that they overtly develop in those muscles which connect the moving segment(s) to the nearest fixation point (Belen’kii et al., 1967; Marsden et al., 1981; Cordo and Nashner, 1982; for a review see Massion, 1992), while they are correctly shaped, but kept subthreshold, in those muscles in which their action is useless (Baldissera et al., 2002). An indirect issue which is strictly correlated to the above observations is that APAs conform to the adequacy of the fixation point, being smaller and smaller or even disappearing when the fixation point does not guarantee the full discharge of the perturbation (Brenière et al., 1987 balance during locomotion; Nouillot et al., 1992 balance during unipedal stance; Dietz and Colombo, 1996 balance in water; Esposti and Baldissera, 2011 balance with two fixation chains).

### Intra-Limb APAs

As explained in the introduction, *intra-limb APAs* are those distributed to muscles of the same limb in which the movement occurs. The first evidence of such APAs dates to 1963, when Hopf and Hufschmidt (1963) observed that the anterior deltoid (AD) activity preceded the voluntary recruitment of BB driving elbow flexion. Later on, Aoki (1991) reported that wrist flexions, performed with the hand prone or supine, were accompanied by APAs in muscles acting at the elbow and that these APAs reverted their pattern in relation to the direction of the movement. In this regard, Chabran et al. (2001), observed also that when providing a support to the elbow the level of postural activity in shoulder muscles was deeply depressed, but the *intra-limb* APAs chronology remained unaffected. Examples of intra-limb APAs were also reported for shoulder and elbow movements by Almeida et al. (1995) and by Gribble and Ostry (1999).

**Figure 1:**
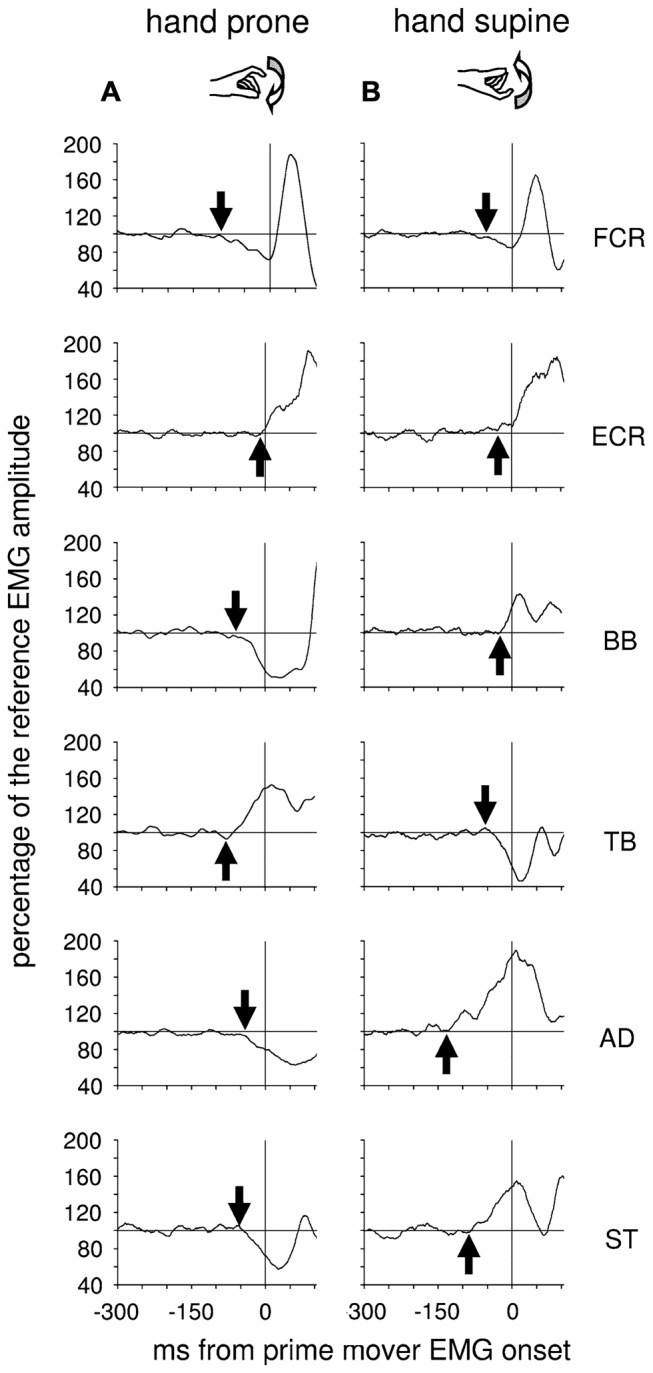
**Postural adjustments in upper-limb muscles preceding an index-finger tap with the prone or supine hand.** Each graph displays the Anticipatory Postural Adjustments (APA) onset (arrow) and its development on the tonic EMG from different postural muscles of a single representative subject, with the hand resting prone **(A)** or supine **(B)**. Postural muscles: flexor carpi radialis (FCR), extensor carpi radialis (ECR), biceps brachii (BB), triceps brachii (TB), anterior deltoid (AD), superior trapezius (ST). The vertical line at 0 ms marks the onset of the prime mover activity. Note that in the muscles acting at the elbow, the shoulder and the trunk APAs reverts in sign when hand posture changes from prone to supine. EMG is rectified, integrated and averaged (75 trials) and its size expressed in percentage of the mean EMG level recorded 1 s before the go signal. *Reproduced from Caronni and Cavallari (2009a), © Springer-Verlag 2008, with permission of Springer*.

**Figure 2:**
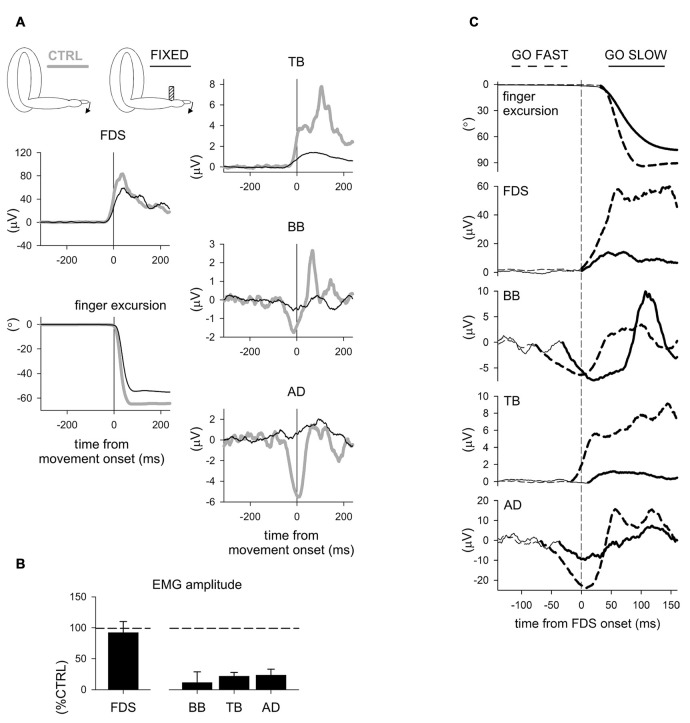
**Effects of fixation point and movement velocity on APAs.** **(A)** Recordings of EMG activity in index-finger prime mover and intra-limb APAs in a representative subject, with or without a wrist fixation point. It is evident that both the EMG activity in the prime mover flexor digitorum superficialis (FDS) and the finger movement were comparable both with (FIXED, black) and without (CTRL, gray) the wrist fixation point, while in the FIXED condition APAs were deeply reduced in BB and TB and completely abolished in AD. **(B)** Mean amplitude of pre-movement FDS EMG and of APAs in BB, TB and AD. Values in % of the control sequence (CTRL). A *t*-test showed no CTRL vs. FIXED difference in FDS activation (*t*_6_ = 0.44, *p* = 0.67). The average inhibitory effects on BB and AD and excitation in TB revealed a significant reduction in the FIXED condition (*t*_6_ = 5.01, *p* = 0.002; *t*_6_ = 12.41, *p* < 0.0001; *t*_6_ = 7.80, *p* = 0.0002, respectively). *Reproduced from Bruttini et al. (2014), © Springer-Verlag Berlin Heidelberg 2014, with permission of Springer*. **(C)** Go-fast and go-slow movements in a representative subject. Goniometric recording of the index-finger flexion (top panel) and rectified and integrated (25 ms) EMG from the prime mover FDS and from BB, TB and AD. Note that when moving as fast as possible (GO FAST, dashed traces) the prime mover onset was preceded by APAs in BB, TB and AD. APAs (emboldened) were instead clearly delayed when moving at ~50% of maximal speed (GO SLOW, solid traces). Time 0 (vertical dashed line) refers to prime mover EMG onset. *Reproduced from Esposti et al. (2015), © Springer-Verlag Berlin Heidelberg 2014, with permission of Springer*.

More recently, Caronni and Cavallari (2009a,b) also reported that when only the index-finger is flexed, an APA chain develops in several upper-limb muscles to stabilize the *segmental* equilibrium of the arm. Indeed, these authors described that with the hand prone a brisk finger flexion was preceded by an excitatory burst in Extensor Carpi Radialis (ECR), Triceps Brachii (TB) and Superior Trapezius (ST), while Flexor Carpi Radialis (FCR), BB and AD showed a concomitant inhibition of their tonic activity (Figure 1A). The ECR-FCR coupled activities were appropriate for contrasting the by-product wrist flexion torque due to the voluntary contraction of the index-finger flexors. At the same time, the TB-BB and ST-AD actions contrasted the elbow and shoulder flexion torques produced by the upward perturbation that the index-finger flexion discharged on the metacarpophalangeal (MP) joint. Interestingly, when the hand posture was changed from prone to supine (Figure 1B), the APA pattern reverted in sign in the elbow and shoulder muscles but not in ECR and FCR. This is consistent with the fact that in this condition the perturbation produced on the MP joint was inverted, i.e., downward, but the by-product of recruiting the index-finger flexors was again a wrist flexion torque. These observations witness that APAs are versatile enough to adapt to the mechanical requirements of the postural context. Another proof that *intra-limb* APAs adapt to changes in the postural context within few trials comes from Bruttini et al. (2014) who illustrated that when tapping with the right index-finger, *intra-limb* APAs were deeply reduced (Figures 2A,B) if the right forearm was fixated at the wrist, i.e., closer to the voluntary moving segment. Moreover, Esposti et al. (2015) confirmed that also the *intra-limb* APAs adapt to changes in movement speed (Figure 2C).

In conclusion, *intra-limb* and *inter-limb* APAs seem to share similar control mechanisms. Indeed, both of them: (1) are distributed to several muscles creating a postural chain aiming to prevent the effects of the interaction torques generated by the voluntary movement; (2) revert in sign when movement direction is reverted; and (3) adapt to changes of the postural context within few trials of movement repetition. Thus, the central nervous system seems to use the same organization of the motor command for controlling both the segmental and the whole-body posture.

## APA and Precision

The idea that the precision of a voluntary movement relies on proper APAs was first proposed in relation to *inter-limb* APAs. When pointing toward targets of different sizes, it has been shown that APAs decreased in size as the accuracy demand increased, i.e., when pointing smaller and smaller targets. This feature has been shown both in the upper-limb (Bonnetblanc et al., 2004; Nana-Ibrahim et al., 2008) and in the lower-limb (Bertucco and Cesari, 2010). Lower limb pointing was also investigated by Duarte and Latash (2007), which have shown that as movement velocity increased, so did APA variability. The relation between movement speed and scattering of the final position around a target was also well described (Schmidt et al., 1979; Fernandez and Bootsma, 2004). All these observations suggest that small and less variable APAs should accompany slow but precise movements. Finally, Berrigan et al. (2006) reported that when pointing is performed towards small targets (i.e., under high accuracy constraints) from an unstable position (i.e., standing vs. sitting), slowing movement speed represents a strategy to reduce the equilibrium disturbance and the associated APAs. However, the above results might simply be an outcome of the relationship between APAs and intended movement speed (Shiratori and Aruin, 2007; Esposti et al., 2015), given the well known inverse relation (Fitts, 1954 law) between target width and movement speed. In order to get rid of such possible bias, Caronni et al. (2013) studied *inter-limb* APAs during an upper limb pointing movement toward a target of fixed dimension, while wearing (or not) prismatic lenses, which shift the binocular eye field and make the subject to miss the target (Redding et al., 2005). Caronni et al. (2013) observed that the focal movement had similar kinematics in the two conditions, but after donning or doffing the lenses appreciable pointing errors occurred. Moreover, when committing such errors, APAs in the lower limb were out of proportion with respect to the recruitment of the upper limb prime mover.

The linkage between APAs and movement precision is also supported by the observation that training induces improvement in the correct tailoring of the APA chain with respect to the prime mover recruitment, both in young adults (Kanekar and Aruin, 2015) and elderly (Kubicki et al., 2012; Aruin et al., 2015). Thus, the increased movement precision observed after training (Hamman et al., 1995; Yang et al., 2013) might be partly due to a more appropriate tuning of the postural adjustments. Other suggestions about the linkage between APAs and precision derive from some motor behaviors in which proper whole-body stabilization is needed to achieve an effective performance. According to shooting coaches and athletes, good postural balance is a vital component of successful shooting. During bipedal standing, top-level rifle shooters stabilized their whole body balance better than naive shooters (Aalto et al., 1990); the capability to reduce the oscillation, especially in the last few seconds before pulling the trigger, expresses better control of posture in athletes and was associated with a better shooting performance (Era et al., 1996; Mononen et al., 2007). More recently, Furuya et al. (2011) demonstrated that professional pianists tended to play using less muscular activity and take greater advantage of shoulder joint rotation during a keystroke than did novice.

**Figure 3:**
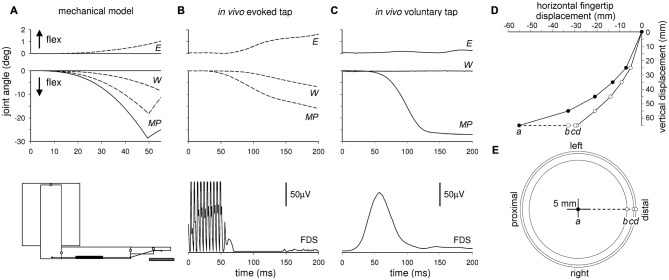
**Comparison of arm joint kinematics during a simulated, an *in vivo* evoked and a voluntary finger flexion.** Time course of a finger flexion at the metacarpophalangeal (*MP*) joint, and the related changes at wrist (*W*) and elbow (*E*) level, measured in an arm mechanical model (bottom-left inset) when the proximal segments were free to rotate **(A**, dashed lines**)** or when they were immobilized **(A**, solid lines**)**, so as to produce fictive APAs. Angular displacements of the three joints were also recorded when an index-finger tap was passively evoked *in vivo* by electrical stimulation of the median nerve **(B)** and when it was voluntary performed **(C)** Rectified EMG activity of prime mover FDS in the two lower graphs. Note that the mechanical model well predicts the displacements of the proximal joints both during voluntary (solid lines) and evoked (dashed lines) index-finger tap. Panel **(D)** illustrates the simulated fingertip trajectory when the proximal segments were immobilized (filled circles, fictive APAs), and when they were free to rotate (empty circles). Note that for a vertical displacement of 65 mm, the fingertip hits the table surface (dashed line) more proximally with fictive APAs (*a*) then without them (*d*). Dots *c* and *b* mark the hitting position when the fictive APAs were restrained to the sole shoulder or to shoulder plus elbow, respectively. In the planar graph **(E)** the filled circle is the univocal target position theoretically resulting from a fully expressed APA control. Any disturbance of the APA chain would necessarily lead to impact any other point. *Reproduced from Caronni and Cavallari (2009a), © Springer-Verlag 2008, with permission of Springer*.

In regard to *intra-limb* APAs, Caronni and Cavallari (2009a) suggested that during a brisk index-finger flexion, *intra-limb* APAs not only would guarantee the maintenance of the arm posture, but should also be very important in controlling the trajectory (Figure 3C) and thus the final position of the moving segment, i.e., what is clinically indicated as *metria*. Indeed, when simulating an index-finger flexion using a four-joint software mechanical model of the arm (Figure 3A), in which only the prime mover was recruited, a clear disturbance of both focal movement and upper-limb posture was observed, with relevant changes at wrist and elbow level. This would affect the final position of the intentional finger movement (Figures 3D,E). In the model, the only way to prevent these *collateral effects* was to block all segments but the finger, preventing the proximal joints from rotating (fictive *intra-limb* APAs).

**Figure 4:**
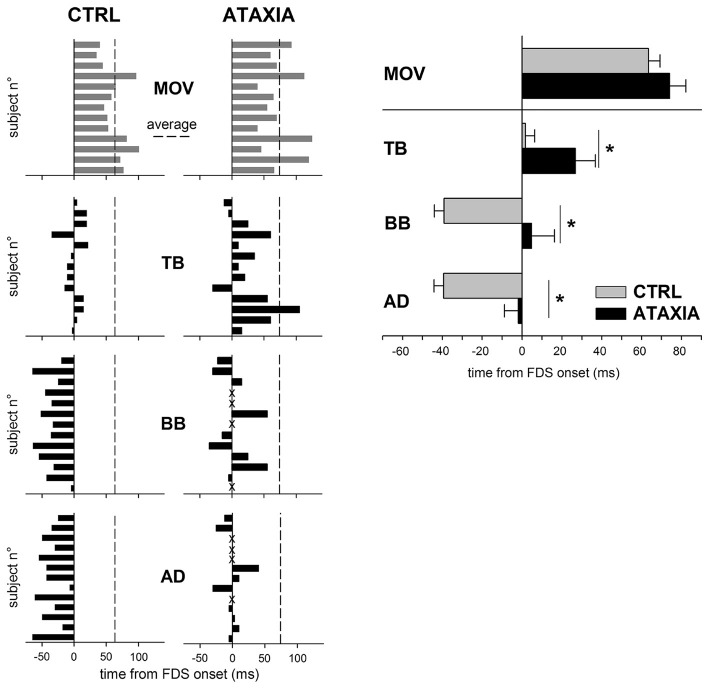
**Comparison of the APA chain in healthy (CTRL) and in cerebellar (ATAXIA) subjects.** Latencies of finger flexion (MOV) and APA onsets in TB, BB and AD are plotted with respect to the onset of FDS, prime mover. Each single subject is represented. Dashed line marks the average movement latency for either group of subjects. Note that in ATAXIA APAs are delayed or, in some cases absent (marked with X). The right panel shows mean latency (± SE) of the onset of finger flexion and APAs. Asterisks mark significant differences found by the unpaired *t*-test. *Reproduced from Bruttini et al. (2015), © Springer-Verlag Berlin Heidelberg 2014, with permission of Springer*.

Since this observation was derived from a very simplified system, Caronni and Cavallari (2009a) also looked for a more realistic situation: a finger tap was electrically evoked in a real arm by stimulating the median nerve (Figure 3B); such an experiment showed recordings comparable in sign and size to those predicted by the software mechanical model, including the dysmetric motor output. However, both the *software simulation* and the *electrically evoked* *tap* paradigms did not faithfully represent the *natural* *dysmetric behavior*, since in the two cases no voluntary command is modeled or generated, respectively. An indirect suggestion of the role of APAs in movement *metria* comes from Bruttini et al. (2015), who demonstrated that when performing a brisk index-finger flexion, cerebellar subjects showed a timing-disruption of *intra-limb* APAs (Figure 4), while their pattern (excitation in TB; inhibition in BB and AD) was unmodified. These data open the question whether cerebellar dysmetria may stem from an erroneous timing of APAs.

**Figure 5:**
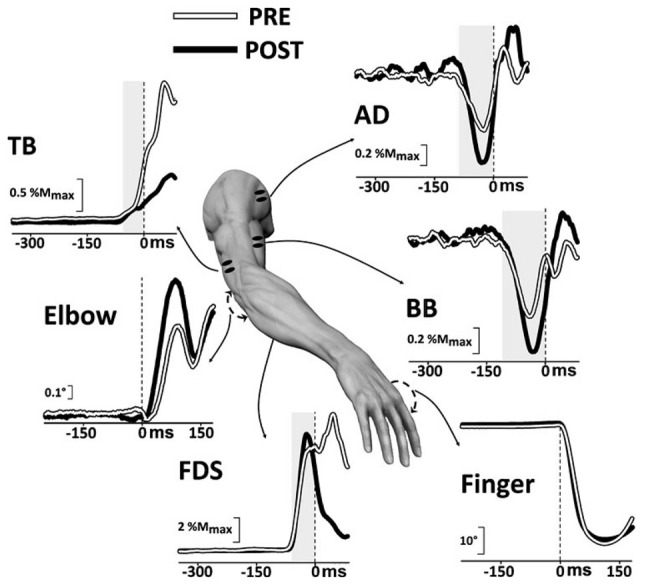
**Intra-limb APAs change after short-term immobilization.** Average recordings in a representative subject before (PRE, white) and after (POST, black) immobilization. When rapidly flexing the index-finger (prime mover FDS), the arm equilibrium is preserved thanks to APAs (shaded area), which are excitatory in TB and inhibitory in BB and AD. After a 12-h immobilization of the wrist and fingers, FDS activation preceding the movement onset and index-finger movement were unchanged. Instead, inhibitory APAs in BB and AD apparently increased, while excitatory APA in TB was marginally decreased. The changes in the postural chain led to a less effective fixation of the elbow joint, which showed a larger displacement during index-finger acceleration. EMG amplitude was normalized to the maximal motor response (*M*_max_) evoked by orthodromic nerve stimulation. *Reproduced from Bolzoni et al. (2012), © Springer-Verlag 2012, with permission of Springer*.

Moreover, considering that a disruption of the APA chain may lead to movement inaccuracy and taking into account that Moisello et al. (2008) demonstrated that 12-h of immobilization are already sufficient in affecting the inter-joint coordination by interfering with the feed-forward mechanisms, Bolzoni et al. (2012) investigated the effect of a short-term immobilization on the *intra-limb* postural control accompanying index-finger flexion. In this article it was shown that 12-h of wrist and finger immobilization effectively modified APAs on elbow and shoulder muscles, without altering the prime mover activation (Figure 5). The APA modifications were also paralleled by a less efficient stabilization of the elbow joint. Therefore, these results may shed light on some of the mechanisms underlying the feeling of motor awkwardness and the reduction of the voluntary movement precision that are commonly experienced after the removal of a cast or a splint.

A last apparent evidence in favor of the importance of APAs in movement precision has been recently provided by Bruttini et al. (2016), who illustrated that the different precision observed when comparing pointing movements performed with the preferred vs. non-preferred hand partly stems from changes in the temporal organization of APAs in the two sides. Indeed, APAs were delayed when moving the non-preferred side. This delay was associated to an impaired stability of the elbow joint during the wrist pointing movement. As a result, the focal movement perturbation caused an increased elbow excursion in the non-preferred hand, eventually leading to the diminished movement precision on that side.

Surely, APAs are not the only determinant of movement precision. In fact, it is well known that when a healthy subject points to a target cross, an eventual error in reaching the target derives from an incorrect sensorimotor transformation, from the visual representation of the target to the kinematics representation of the planned trajectory (Soechting and Flanders, 1989a, b; for a review see also Massion, 1992). Indeed, these authors showed that when subjects have to reach a position which has been previously appreciated kinesthetically (thus after having empirically built up the exact transformation), pointing errors dramatically reduce. In particular, several studies showed that the parietal cortices play a critical role in integrating visual and somatic inputs for building up this sensorimotor transformation. Such transformation seems also assisted by the skin receptors, which detect the torsion forces that act on the skin of the feet in contact with the soil (for a review see Kalaska et al., 1997). In this context, it has been also observed (Esposti et al., “APAs associated to reaching movements are programmed according to the availability of visual information”, submitted manuscript) that once the target position has been acquired, both visually and kinesthetically, reaching the target while gazing it or in a *blind* condition resulted in similar levels of pointing accuracy; pointing precision was instead significantly impaired in absence of visual information. Finally, these authors suggested that the visuospatial memory may play a key role in movement accuracy, while “active vision” seems to be more engaged in movement precision.

## Central Organization of APAs

### Anatomical and Physiological Data

**Figure 6:**
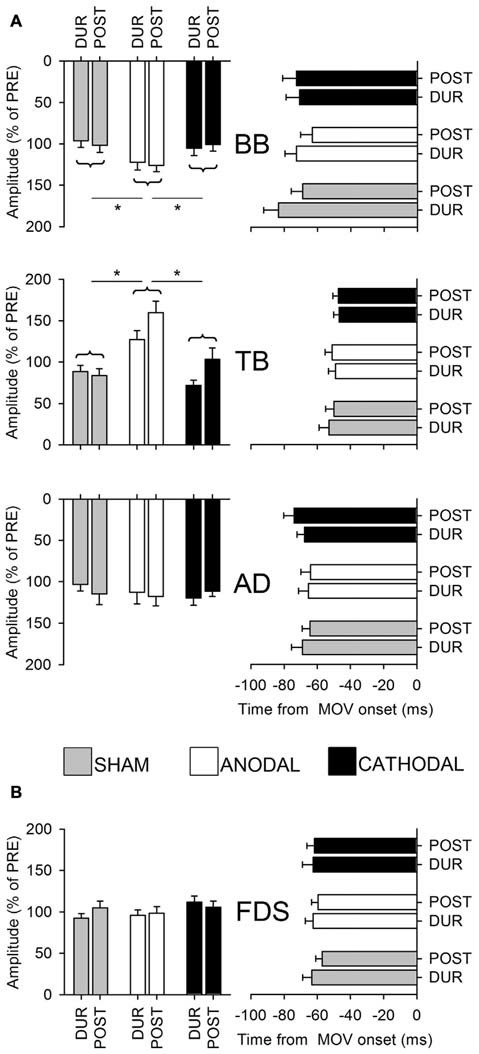
**Amplitude and latency of intra-limb APAs (A) and of voluntary recruitment of index-finger prime mover (B)**. Postural muscles: BB, TB and AD, prime mover FDS. Data were collected from the whole population, in the last 5 min of 1 mA Transcranial Direct Current Stimulation (tDCS) application (DUR) and 20 min after its end (POST). Amplitude data are plotted on the left (expressed as percentage of the amplitude measured before tDCS, % of PRE), latencies referred to movement onset on the right (mean ± SE). Repeated measures *stimulation × time* ANOVA, and Tukey *post hoc* (see asterisks), found a significant increase in the amplitude of BB and TB APAs under ANODAL vs. CATHODAL and SHAM stimulation, while the APA in AD was unaffected. No effect of *time* or *interaction* was observed, thus ANODAL stimulation enhanced APAs in TB and BB with an effect lasting at least 20 min. No significant changes were observed in APAs latency. Note also that tDCS had no effect on the amplitude and latency of FDS activity, witnessing that the increase in BB and TB APAs should not be ascribed to a stronger recruitment of the prime mover. *Reproduced from Bolzoni et al. (2015), © 2015 Elsevier B.V., with permission of Elsevier*.

Several studies support the idea that *Supplementary Motor Area* (SMA) is involved in APAs generation. Severe APA impairments were observed in the bimanual load lifting (the so called *barman task*) when the load was held with the forearm contralateral to the lesioned SMA while no APA changes were observed in a patient with normal SMA, but suffering a complete callosal section (Massion et al., 1989; Viallet et al., 1992). This finding supports the idea that coordination between the posture and movement travels through subcortical level. Moreover, a 1-Hz repetitive Transcranial Magnetic Stimulation (TMS) on SMA, which induces an inhibitory effect, reduces the duration of APAs prior to stepping, without affecting their peak amplitude (Jacobs et al., 2009). More recently, Transcranial Direct Current Stimulation (tDCS) over the SMA has been shown to elicit a differential effect on the postural and the focal component of the movement (Figure 6), supporting the involvement of SMA in APA programming (Bolzoni et al., 2015).

**Figure 7:**
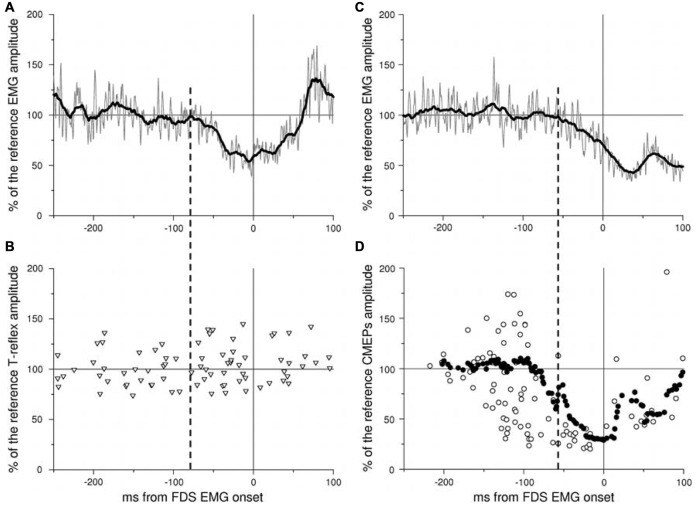
**Time course of spinal and cortico-spinal excitability in the resting BB before an index finger flexion.** An inhibitory APA (iAPA) occurs in the BB EMG in two representative subjects **(A,C)**: in both cases rectified (gray trace) and integrated (black trace) EMG activity (average of 40 trials) is strongly reduced well in advance to the onset of FDS, taken as time 0. The time course of BB spinal excitability has been tested by means of tendon tap reflexes **(**T-reflex, triangles in **B)**, while that of BB cortico-spinal excitability by Transcranial Magnetic Stimulation (TMS)-elicited cortical motor-evoked potentials **(**CMEPs, open circles in **D)**. Each symbol marks amplitude and latency of one single evoked potential. Note that T-reflexes are un-modulated in size in the iAPA window, edged by the iAPA onset (vertical dashed line) and prime mover onset (vertical continuous line), whereas CMEPs are strongly inhibited. To better identify the onset of this inhibitory effect, the CMEPs time course has been integrated with the same time constant used for the EMG (filled circles). Both EMG and evoked potential amplitude are expressed as a percentage of their mean reference amplitude, i.e., that measured before the acoustic go signal. *Reproduced from Caronni and Cavallari (2009b), © Springer-Verlag 2009, with permission of Springer*.

The role of the *Primary Motor Cortex* in generating APAs has been shown by both human and animal studies. The stimulation of the primary motor cortex in the intact cats was indeed able to evoke movement in the contralateral side and APAs in the supporting limbs (Gahéry and Nieoullon, 1978), suggesting that the primary motor cortex in cats is able to control both the voluntary prime mover and the associated postural adjustments. Moreover, in cats, the dynamic of the discharge frequency in pyramidal neurons is time locked and directly proportional with the center of pressure displacement, a key parameter associated to APAs preserving the whole body equilibrium (Yakovenko and Drew, 2009). The role of the primary motor cortex in the anticipatory postural control was also observed in human studies. Indeed, Palmer et al. (1994) used the TMS to induce a silent period in either the left or the right M1 while the subject abducted his left arm, a movement which is known to be preceded by APAs in the contralateral Latissimus Dorsi muscle. Left M1 stimulation produced a delay of the APA onset, while the prime mover timing was unmodified; instead, right M1 stimulation just delayed the prime mover activation. Moreover, it has been shown for both *inter-* (Petersen et al., 2009) and *intra-limb* APAs (Caronni and Cavallari, 2009b) that when a postural muscle is at rest the spinal excitability is un-modulated during the whole period of motor preparation, while when that muscle is recruited in a postural chain the cortical motor action potentials are modulated according to the time course of APAs (Figure 7). From one perspective, this indicates that APAs processing involves M1 level, and from another this suggests that APAs and voluntary commands cannot be decoupled, since the former remains subliminal, i.e., it does not produce a mechanical effect, when the muscle is at rest. Although this may appear uneconomical, this result corroborates two previous observations in which the cortical excitability of hand movers has been shown to fluctuate under the threshold for motoneuronal firing when the ipsilateral foot was voluntarily oscillated (Baldissera et al., 2002) and in which the *hidden effect* developed in an overt APA when the hand was recruited in a postural act (Baldissera and Esposti, 2005).

Regarding *Basal Ganglia*, severe APAs impairment in patients with Parkinson’s disease were already observed by Viallet et al. (1987). More recently, anticipatory brain activity associated to a bimanual load-lifting task was localized in basal ganglia, SMA and thalamus, contra lateral to the load-bearing arm (Ng et al., 2013). It is worth noting that these areas are component nodes of the basal ganglia-thalamo-cortical motor network, which is known to be implicated in well-learned finger movements (Boecker et al., 1998). This indicates a superposition of the neural structures for APAs and those for voluntary motor command, and indirectly supports the view of a oneness of the motor command for both posture and primary movement.

The *Cerebellum* is deeply involved in APA regulation. Babinski (1899) reported that a cerebellar lesion disrupted the coordination between voluntary movement and equilibrium stabilization, indicating that the cerebellum is involved in postural control. This view agrees with the idea that the cerebellum contains forward internal models that could predict the consequences of an action (according to the perceived postural context) and can be used to overcome time delays associated with feedback control (Wolpert et al., 1998; Imamizu et al., 2000). Several studies on patients positively concluded for such an involvement. Indeed, patients suffering cerebellar lesions failed to show a normal anticipatory adjustment in grip force when lifting or moving an object (Müller and Dichgans, 1994; Babin-Ratté et al., 1999) and cerebellar lesions abolished APAs plasticity in a bimanual unloading task (Diedrichsen et al., 2005). Congruently, Diener et al. (1992) reported that cerebellar patients produce a normal pattern of APAs, but with abnormalities in their timing relationship with the onset of the prime mover. Finally, as shown in Figure 4, Bruttini et al. (2015) demonstrated that when performing a brisk index-finger flexion, cerebellar subjects showed a timing-disruption of *intra-limb* APAs, while their pattern was unmodified. Using functional magnetic resonance imaging, Schmitz et al. (2005) reported that APAs were associated with activation of sensorimotor areas, SMA and the cerebellum. On the contrary, Ng et al. (2013) found no evidence of cerebellar involvement during APAs using magnetoencephalography in a bimanual coordination task. Further support on the role of cerebellum in timing APAs come from animal studies. When reaching a water flask with the mouth, wild type mice showed a clear APA in hind-limb muscles, synchronous to neck muscles, while in transgenic mice with defective cerebellar Purkinje cells the hind-limb activity occurred markedly later than that in neck muscles (Yamaura et al., 2013).

Finally, Schepens and colleagues (Schepens and Drew, 2004; Schepens et al., 2008) emphasized the role of pontomedullary reticular formation (PMRF) in the coordination of posture and movement. In particular, they suggested that PMRF is a site of integration of signals from both cortical and subcortical structures, and that these signals ensure that APAs are appropriately scaled in time and magnitude to the intended movement, contributing to integrate the control of posture and movement, as also illustrated by Toussaint et al. (1998).

In summary, these anatomical and physiological studies show a large superimposition between neural structures governing the voluntary and the postural components of the movement, leaving the question open on whether these two processes are implemented separately or they are an expression of a unique *posturo-focal* command.

### Dual vs. Shared Command Hypothesis

**Figure 8:**
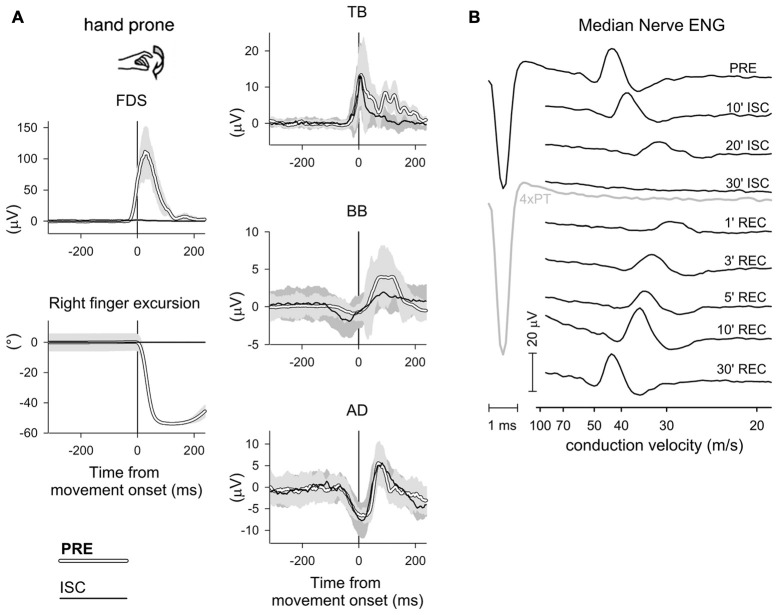
**APA persistence under ischemia.** **(A)** Rectified and integrated average recordings of index-finger prime mover EMG and of intra-limb APAs, before and during ischemic block of the median nerve at the forearm. One representative subject. Time 0: movement onset of the left index-finger. When rapidly flexing both index-fingers before ischemia (PRE, white traces, SD in light gray), the right prime mover FDS was activated and the right arm equilibrium was preserved, thanks to an excitatory APA in TB and inhibitory APAs in BB and AD. Under ischemia of the right forearm (ISC, black traces, SD in dark gray), FDS activation and index-finger movement were both suppressed, while excitatory APAs in TB and inhibitory APAs in BB and AD were still evident and qualitatively similar to those recorded in PRE. **(B)** Electroneurogram (ENG) of the right median nerve of one representative subject, showing the effect of ischemia, induced by a pressure cuff, on the afferent volley elicited by electrical stimulation of the index-finger skin (3 × perception threshold, PT). Ischemia progressively reduced the volley amplitude until reaching complete suppression after 30 min (even increasing stimulation to 4 × PT, gray trace, did not elicit a response). After removing the pressure cuff, the effect of ischemia completely recovered in 30 min. *Reproduced from Bruttini et al. (2014), © Springer-Verlag Berlin Heidelberg 2014, with permission of Springer*.

According to the classical view, the prime mover activity and the associated postural adjustments result from two different central commands *(dual command hypothesis)*, which are independently dispatched to the prime mover and to the muscles generating the postural chain (Babinski, 1899; Hess, 1943; Cordo and Nashner, 1982; Brown and Frank, 1987). Conversely, a growing body of evidences favors the view that APAs and prime mover recruitment are both controlled by a *shared motor command* (Aruin and Latash, 1995; Stapley et al., 1998, 1999; Caronni and Cavallari, 2009b; Petersen et al., 2009). In this regard, it is interesting to mention the studies of Gritsenko et al. (2009) and Leonard et al. (2011), showing that when correcting an ongoing arm pointing movement, the CNS employs a predictive mode of postural control and consistently adapts the postural muscle activities before modifying the prime mover recruitment. These authors concluded that the postural corrections could be described as being a component of the voluntary movement, rather than ensuring the maintenance of equilibrium. Further, Bruttini et al. (2014) demonstrated that the APA chain associated to a voluntary movement cannot be decoupled from the command driving the focal movement. Indeed, when repeatedly trying to flex the index-finger under forearm ischemia, so that the voluntary command was normally dispatched but the prime mover not able to receive it, significant anticipatory adjustments were clearly visible in BB, TB and AD (Figure 8).

In this peculiar condition, in which no postural perturbation is generated, one would have expected that APAs were suppressed since unnecessary and uneconomical, unless the postural and the prime mover muscles obey a “shared” motor command. It is of course apparent that “ischemia” results does not allow to discern whether: (i) one specific neural structure generates both the postural and the voluntary commands; or (ii) two neural structures exist, one for the voluntary and the other for the postural commands, and one of the two acts as a master on the other; or (iii) a third neural structure exists, which simultaneously triggers the two slave “postural” and “voluntary” neural structures. However, from the point of view of the muscles (i.e., the only aspect that such experimental approach allowed to observe), these three ways of organizing the posturo-focal integration are all identical. Given this neural organization, it may be conceived that the system prefers an *economy* in computational terms, rather than in diminishing the number of activated neurons. In this case, one may imagine that any given voluntary command is associated with an arborized pattern of postural commands, forwarded to a number of possible fixation points. Thus, the most useful arborization for providing the actual support would be supraliminarly recruited, while the leftover branches would be still present but kept silent, i.e., under threshold. The amplification of APA transmission to certain targets, and the parallel attenuation towards other targets, could be automatically accomplished by servo-systems like PMRF neurons. This would effectively free the upper centers from the computational demand of adapting the APAs to the postural context. This view is also corroborated by the already cited works of Baldissera et al. (2002) and Baldissera and Esposti (2005), who showed that subliminal *inter-limb* APAs in a resting arm during foot movements become supraliminal when that segment is actively used for postural stabilization. Similar results were also shown for *intra-limb* APAs by Caronni and Cavallari (2009b).

The oneness of postural and voluntary command is also supported by the finding that the APA latency depends on the movement instruction, not on its actual velocity (Esposti et al., 2015). This conclusion was based on two observations. First, no correlation was found between APA latency and movement speed when subjects had to follow a *go fast* instruction (i.e., to move as fast as possible), nor when they had to *go slow* (at half their maximal speed). Second, the maximal speed was variable among subjects, so that minimum movement speeds within the range of *go fast* trials intermingled with maximal movement speeds within the range of *go slow* trials. Despite similar velocities, APAs were earlier in the former and less anticipated in the latter. Thus, APAs looked much more tailored on the expected perturbation than on the real one, favoring the idea of a functionally unique postural and focal command.

## Conclusions

This review shows that *intra-limb* APAs and *inter-limb* APAs share the same organizational and behavioral principles. The simplicity and flexibility of the *finger tap* experimental model has also allowed to shed light on a new general characteristic of the postural control. In fact, APAs not only deal with whole-body equilibrium, segmental posture and movement initiation, but are also involved in providing the correct postural set necessary to obtain precise movements. Finally, this approach has been useful in inspecting the central organization of APAs. In particular, the persistence of APAs after the ischemic block of the prime movers provides a principal piece of evidence in assessing the oneness of the focal and postural commands.

## Author Contributions

All authors contributed in collecting the literature, critically analyzing it and writing the manuscript. All authors approved the final version and agree to be accountable for all aspects of this work.

## Conflict of Interest Statement

The authors declare that the research was conducted in the absence of any commercial or financial relationships that could be construed as a potential conflict of interest.
